# Outcomes and Adverse Effects of Deep Brain Stimulation on the Ventral Intermediate Nucleus in Patients with Essential Tremor

**DOI:** 10.1155/2020/2486065

**Published:** 2020-08-01

**Authors:** Guohui Lu, Linfeng Luo, Maolin Liu, Zijian Zheng, Bohan Zhang, Xiaosi Chen, Xing Hua, Houyou Fan, Guoheng Mo, Jian Duan, MeiHua Li, Tao Hong, Dongwei Zhou

**Affiliations:** ^1^Department of Neurosurgery, The First Affiliated Hospital of Nanchang University, Nanchang, Jiangxi, China; ^2^The First Clinical Medical College of Nanchang University, Nanchang, Jiangxi, China; ^3^Queen Mary College of Nanchang University, Nanchang, Jiangxi, China

## Abstract

**Objective:**

This study was aimed at identifying the potential outcome predictors, comparing the efficacy in patients with different tremor characteristics, and summarizing the adverse effect rates (AERs) of deep brain stimulation on the ventral intermediate nucleus (VIM-DBS) for essential tremor (ET).

**Methods:**

An extensive search of articles published to date in 2019 was conducted, and two main aspects were analyzed. Improvement was calculated as a percentage of change in any objective tremor rating scale (TRS) and analyzed by subgroup analyses of patients' tremor characteristics, laterality, and stimulation parameters. Furthermore, the AERs were analyzed as follows: the adverse effects (AEs) were classified as stimulation-related, surgical-related, or device-related effects. A simple regression analysis was used to identify the potential prognostic factors, and a two-sample mean-comparison test was used to verify the statistical significance of the subgroup analyses.

**Results:**

Forty-six articles involving 1714 patients were included in the meta-analysis. The pooled improvement in any objective TRS score was 61.3% (95% CI: 0.564-0.660) at the mean follow-up visit (20.0 ± 17.3 months). The midline and extremity symptoms showed consistent improvement (*P* = 0.440), and the results of the comparison of postural and kinetic tremor were the same (*P* = 0.219). In addition, the improvement in rest tremor was similar to that in action tremor (OR = 2.759, *P* = 0.120). In the simple regression analysis, the preoperative Fahn-Tolosa-Marin Tremor Rating Scale (FTM-TRS) scores and follow-up time were negatively correlated with the percentage change in any objective TRS score (*P* < 0.05). The most common adverse event was dysarthria (10.5%), which is a stimulation-related AE (23.6%), while the rates of the surgical-related and device-related AEs were 6.4% and 11.5%, respectively.

**Conclusion:**

VIM-DBS is an efficient and safe surgical method in ET, and the efficacy was not affected by the body distribution of tremor, age at surgery, and disease duration. Lower preoperative FTM-TRS scores likely indicate greater improvement, and the effect of VIM-DBS declines over time.

## 1. Introduction

Essential tremor (ET), also known as primary tremor, is defined as an isolated tremor syndrome consisting of a bilateral upper extremity action tremor for at least 3 years with or without tremor in other locations and without other neurological signs [1]. Currently, the management of this disorder focuses on controlling the symptoms, and pharmacotherapy is the primary therapy. Unfortunately, drug therapy is only effective in 50% of ET patients [2]. Surgical options include stereotactic radiofrequency thalamotomy, gamma knife thalamotomy, and deep brain stimulation [3–5] Among these options, deep brain stimulation in the ventral intermediate nucleus (VIM-DBS) is more easily reversed than thalamotomy and can effectively suppress tremors while avoiding the common complications of thalamotomies [6, 7]. The posterior subthalamic area/caudal zona incerta and subthalamic nucleus, except for the VIM, are also targets of DBS; however, thus far, studies are still limited with a short follow-up period compared to that in studies investigating VIM [8].

Although the effect of DBS on essential tremor is definitive, several factors influence the therapeutic effect. As reported by Putzke et al., the significant predictive factors associated with increased tremor severity at the initial clinical visit include an older age and a longer disease duration [9]. In addition, in most cases of ET, the tremor score worsens over time, and the average tremor severity increases each year [10]. Previous studies have found that the benefits of DBS are affected by laterality and stimulation parameters [11, 12]. Ondo et al. concluded that unilateral thalamic DBS is less effective than bilateral DBS in controlling appendicular and midline ET [11]. Moreover, a previous study found that to optimize tremor control, the stimulation parameters, including the voltage, frequency, and pulse width, need to be adjusted [12].

Similar to all surgical interventions, DBS may cause potential perioperative and postoperative adverse effects (AEs), such as infection, hemorrhage/hematoma, and pneumonia [13], affecting the prognosis of many patients. Therefore, further analysis of AE rates (AERs) is urgently needed. A large meta-analysis is also imperative to provide a comprehensive assessment of the prognostic factors, safety, and efficacy of VIM-DBS in the treatment of ET.

## 2. Methods

### 2.1. Search Strategy

**Figure 1:**
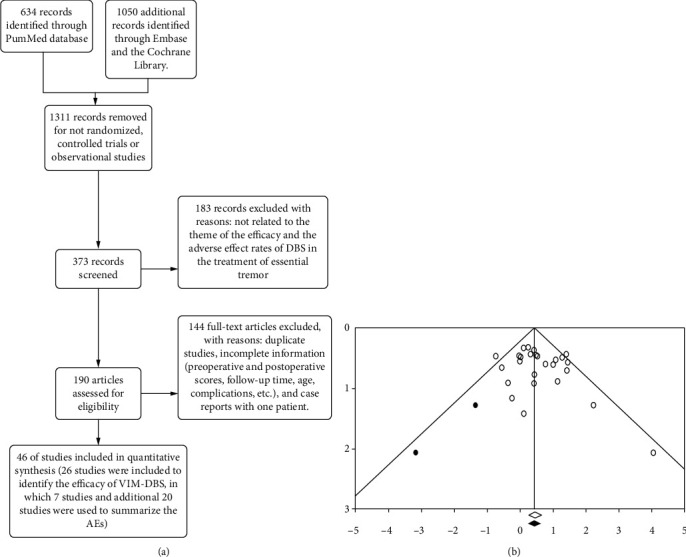
The PRISMA flowchart and funnel plot: (a) the PRISMA flowchart; (b) the funnel plot of the studies evaluating TRS scores. The plot shows an equal distribution of studies and has no presence of bias.

Three electronic databases (PubMed, Embase, and the Cochrane Library) searched following the Preferred Reporting Items for Systematic Reviews and Meta-Analyses (PRISMA) guideline. We searched all articles related to DBS treatment for ET. We did not limit the age, sex, or operative time. A flow chart of the literature search is shown in Figure 1(a). We searched the literature using the keywords “essential tremor”, “ventral intermediate nucleus”, “deep brain stimulation”, and “adverse effect”. In addition, we registered the protocol of this meta-analysis in PROSPERO under the number CRD42020147313.

### 2.2. Inclusion Criteria and Exclusion Criteria

The inclusion criteria for eligible studies were as follows: (1) the study subjects were ET patients treated with VIM-DBS; (2) the studies were randomized, controlled trials or observational studies published in English; (3) the studies reported any objective Tremor Rating Scale (TRS) scores at baseline and the last follow-up visit to determine the efficacy of VIM-DBS; (4) the studies specified the number of AEs in the ET patients; and (5) the studies described the tremor characteristics, such as midline (head/voice) tremor, extremity (arms/legs) tremor, rest tremor, or action (postural and kinetic) tremor. Regarding the efficacy of DBS, the studies had to meet criteria (1), (2), and (3), but the other criteria were optional. Regarding the adverse effects, the studies had to meet criteria (1), (2), and (4), but the other criteria were optional.

Conference presentations, editorials, reviews, non-English studies, and duplicate publications were excluded.

Controlled studies that included cohorts subjected to different surgical procedures were regarded as studies involving separate cohorts. For example, if a study included two cohorts that compared DBS and lesion surgery, the cohorts undergoing DBS were included in our study, and the other cohorts were excluded. Not all included studies performed follow-up evaluations or recorded the mean age, laterality, and stimulation parameters; hence, only studies that reported the same information could be combined for the data analysis. For instance, 46 original studies were included in our study, but only 13 studies reported the pulse width, and we combined these 13 studies for the statistical analysis.

### 2.3. Data Extraction

A data extraction template was used to build an evidence table that included the following items: author, publication year, number of patients, age, duration of disease in years, stimulation site, follow-up time, laterality (right, left, or bilateral), stimulation parameters (pulse width and voltage), any objective TRS scores (mainly including the Fahn-Tolosa-Marin Tremor Rating Scale [14], Essential Tremor Rating Assessment Scale, and Modified Unified Tremor Rating Scale) at baseline and the last follow-up visit, tremor characteristics (midline (head/voice) tremor, extremity (arms/legs) tremor, rest tremor, and action tremor (postural and kinetic)), and number of postoperative AEs.

Two authors (Luo Linfeng and Liu Maolin) independently extracted the data. If there was any disagreement or doubt, consensus was reached by consulting a third party (Lu Guohui).

### 2.4. Statistical Analysis

The statistical analysis was performed using Comprehensive Meta-Analysis (CMA) software (version 3.3.070) and Stata/SE 12.0. A meta-analysis of proportions was performed [15], and only 26 studies in Table 1 were included in the test of heterogeneity. The *I*^2^ value and *Q* statistic were evaluated. If *I*^2^ ≥ 50%, a random effects analysis using the DerSimonian-Laird model was employed to pool the data. Otherwise, a fixed effects model was used. The primary outcome was improvement, which was calculated as a percentage of change in any objective TRS scores [16], and the safety of DBS for ET was evaluated mainly based on adverse events of particular interest, such as dysarthria, paresthesia, hemiparesis/paresis, and headache.

To detect significant differences in the baseline characteristics shown in Table 2 and compare all subgroup analyses of patients' tremor characteristics, laterality, and stimulation parameters, two-sample mean-comparison tests were performed in Stata/SE 12.0, which could calculate the *P* values based on the mean, standard deviation, and sample size. In addition, the potential predictive factors of the percentage of change in any objective TRS score were tested using a simple regression analysis in CMA software [17], and *P* ≤ 0.05 was defined as statistically significant. Publication bias was assessed using a funnel plot (Figure 1(b)) and Begg's test.

Regarding the efficacy of DBS, subgroup analyses were performed according to laterality (unilateral vs. bilateral) [18] and stimulation parameters (voltage and pulse widths) [19–21]. After we identified that the follow-up time is a predictive factor, the data from Barbe et al. [22] were excluded because the patients underwent the operation at least 3 months before their trials to optimize the efficacy of DBS, causing strong heterogeneity based on the sensitivity analysis, and we could not identify the detailed follow-up time after the first surgery.

### 2.5. Quality Evaluation

Two examiners independently conducted a review of the literature to eliminate bias. We used the Newcastle-Ottawa Scale [23] to assess the quality of the nonrandomized studies, including the following evaluations: adequacy of the case definition, representativeness of the cases, selection of controls, definition of controls, comparability of cases/controls, the same method of ascertainment, and nonresponse rate (Table 3). The Newcastle-Ottawa Scale is an easy-to-use, convenient tool for quality assessment, and the total score ranged from 0 (lowest quality) to 8 (highest quality), with one star representing one point. A study with 6 or more stars was classified as a high-quality study.

## 3. Results

### 3.1. Search Results

In total, 46 studies involving 1714 patients were assessed for eligibility by reviewing the full text of the articles. After excluding the articles that did not conform to the eligibility criteria, 4 randomized controlled trials (RCTs) and 42 observational studies were included (Figure 1). Moreover, the 26 studies shown in Table 1 were included to identify the efficacy of VIM-DBS; additionally, 7 of these studies and the 20 additional studies shown in Table 4 were used to summarize the AEs.

### 3.2. Outcome Results

#### 3.2.1. Overall

In total, the 26 included studies involved 439 patients (Table 1). The percentage change in any objective TRS score in all included studies was 61.3% (*P* < 0.001).

#### 3.2.2. Subgroup Analysis of Laterality

To compare the outcomes of DBS treatment with unilateral and bilateral procedures, a subgroup analysis was performed based on laterality. Nine studies involving 165 patients were included in the unilateral procedure group, while six studies involving 72 patients were included in the bilateral procedure group. The unilateral and bilateral improvement was 57.6% and 67.8%, respectively; moreover, the efficacy did not significantly differ between the unilateral and bilateral procedure groups (*P* = 0.139). In addition, the baseline characteristics did not significantly differ between the unilateral and bilateral procedures (Table 2).

#### 3.2.3. Subgroup Analysis of the Stimulation Parameters (Pulse Width and Voltage)

The pulse width data were divided into 60-90 *μ*s (125 patients) and 90-120 *μ*s (142 patients). The improvement in these two subsets was 68.4% (60-90 *μ*s) and 60.2%, respectively (90-120 *μ*s) (*P* = 0.164). Then, the voltages were classified into the following two groups: ≥3.5 V (61 patients) and <3.5 V (236 patients). The improvement by voltages was 61.7% (<3.5 V) and 69.3% (≥3.5 V) (*P* = 0.272). The effect of VIM-DBS was not affected by the stimulation parameters (*P* > 0.05). In addition, the age at surgery and baseline characteristics did not significantly differ between the subgroups (Table 2).

#### 3.2.4. Subgroup Analysis of the Tremor Characteristics

On the one hand, in total, 52 ET patients were included in the analysis of midline (head/voice) and extremity (arms/legs) tremor. However, the improvement in midline and extremity symptoms did not significantly differ (OR = 0.716, 95% CI: 0.307-1.670; *P* = 0.440). On the other hand, in total, 45 ET patients were included in the analysis of rest tremor and action tremor, and the improvement in rest tremor did not significantly differ from that in action tremor (OR = 2.759, 95% CI: 0.768-9.913; *P* = 0.120). Action tremor was divided into postural and kinetic tremor, and in total, 107 patients were included in this subgroup analysis (postural action: 52 patients, kinetic action: 55 patients). The improvement in the group with postural tremor (94.2%) was higher than that in the group with kinetic tremor (46.5%), but there was no statistical significance (*P* = 0.219). All detailed data are shown in Supplementary 1, 2, and 3.

#### 3.2.5. Outcome Predictive Factors

**Figure 2:**
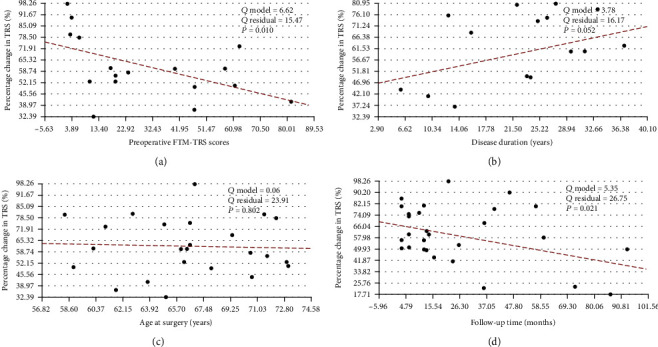
Potential predictive factors for percentage change in any TRS score (%). There were no significant correlations between percentage change in any TRS score (%) and (c) age at surgery (*P* = 0.052) as well as (b) disease duration (*P* = 0.802). There were significant negative correlations between percentage change in any TRS score (%) and a preoperative FTM-TRS score (*P* = 0.010) as well as (d) follow-up period (*P* = 0.021); dots: each study mean percentage change in any TRS score (%); red line of dashes: linear regression line; TRS: Tremor Rating Scale.

To identify the potential outcome predictors, the clinical and demographical factors were tested separately. As shown in Figure 2, the preoperative FTM-TRS scores (*P* = 0.010) and follow-up period (*P* = 0.021) were significantly negatively correlated with the clinical outcomes. There were no significant correlations between the outcomes and other continuous clinical variables, such as age at surgery (*P* = 0.802) and disease duration (*P* = 0.052).

#### 3.2.6. Publication Bias

A funnel plot of the comprehensive outcomes of 26 studies was drawn, and Begg's test found no significant publication bias (*P* = 0.261).

#### 3.2.7. Common Adverse Effects

The frequent events are summarized in Supplementary 4. The incidence of stimulation-related AEs (23.6%) was higher than the incidence of device-related AEs (11.5%) and the incidence of surgical AEs (6.4%). The most common stimulation-related AEs were dysarthria (10.5%), paresthesia (6.3%), hemiparesis/paresis (6.3%), and headache (6.7%).

Rare events were classified as miscellaneous, and the specific details are shown in Supplementary 5.

## 4. Discussion

Our study provides the largest systematic review based on a large sample size, i.e., 46 studies involving 1714 patients, to summarize the efficacy and adverse effect rates of DBS for the treatment of essential tremor. The evidence provided in our meta-analysis shows that DBS targeting the VIM is effective in the treatment of ET, with a pooled improvement of 61.3% in any objective TRS score at 20.0 ± 17.3 months. In addition, our simple regression analysis indicated that the preoperative FTM-TRS scores and follow-up time likely predict the clinical outcomes. The most common adverse event was dysarthria, which is a stimulation-related AE. Based on the results of our study, it is possible to identify patients who are most likely to benefit from this surgical procedure and ultimately improve the quality of life of these patients.

### 4.1. Analysis of Subgroups

In our analysis, the efficacy in rest tremor was not more significant than that in action tremor. Two studies included in the analysis showed 100% improvement, although a ceiling effect may exist [24]. A previous publication reported that the efficacy in terms of tremor of action/intention declined and was less stable over time, while the effect on resting tremor showed limited change [25]. Moreover, Morishita et al. [26] stated that the microlesion effect did not affect resting tremor and, thus, showed a sustained improvement at 6 months after DBS, although the mechanisms leading to the significant improvement in resting tremor are unclear in advancing disease. One study stated that bilateral electrolytic lesions in the cerebellar dentate and interpositus nuclei resulted in tremor at rest [27]. The VIM, which is a target in the surgical treatment of ET, receives cerebellar afferents, and this surgery results in improvement in rest tremor in ET [28]. Hence, VIM-DBS could also be an effective strategy for ET patients with rest tremor.

In accordance with the anatomical distribution of tremor, our results revealed that similar improvements were observed in midline and extremity tremor. In a study conducted by Putzke et al., midline tremor showed significant improvement compared with baseline tremor, while head and voice tremor showed the most consistent improvement [29]. However, the effects on head tremor have been inconsistent according to an analysis conducted by Moscovich et al. [20]. Relatedly, the effect of thalamic stimulation on midline tremor tends to increase with symptom severity [29]. The significant effect of the stimulation on extremity tremor was maintained for 1 year, but the voltage had to be increased in a European trial [30]. Furthermore, it has been reported that midline tremor, including head and voice tremor, showed greater improvement after a bilateral procedure because of the bilateral innervation of neck muscles [29–31]. However, unilateral stimulation is equally effective in the treatment of contralateral hand tremor [32]. Hence, after a series of stimulation adjustments, the second implantation, and short follow-up in the included studies, the improvement in midline and extremities showed no significant difference.

### 4.2. Predictive Factors

Much attention has been paid to the clinical factors that may predict outcomes in patients undergoing DBS for tremor, while a few studies identified potential prognostic factors. It is important for clinicians to evaluate the variables that may influence the clinical outcomes of surgery and predict the therapeutic effects of surgery as accurately as possible [33]. In our simple regression analysis, we concluded that lower preoperative scores indicated greater improvement and that the effect of VIM-DBS declines over time based on 439 ET patients.

A published study retrospectively investigated the clinical features of tremor, including Parkinson's disease, essential tremor, cerebellar tremor, and dystonic tremor, that might predict the outcome of DBS and reported that patients with higher baseline scores had a greater DBS response [34]. Nevertheless, other recognized publications showed that a higher preoperative tremor severity predicted a worse outcome [35, 36]. According to Blomstedt et al. [37], ET patients with a more severe tremor might produce a higher level of residual tremor upon stimulation after surgery, resulting in a worse outcome. Several studies have described the loss of efficacy during a long follow-up following DBS among ET patients [12, 38, 39]. For instance, Paschen et al. concluded that the tremor severity and effect of VIM-DBS significantly deteriorate over a decade in ET patients [38]. A combination of factors has been proposed for the loss of the clinical efficacy of VIM-DBS in ET, including natural disease progression [25, 40], tolerance [25, 41], suboptimal electrode placement [42], increased impedance in brain tissue over time [7], loss of the microthalamotomy effect [7], and long-term, stimulation-induced effects [39]. However, tolerance and the natural progression of the disease are considered the most possible explanations for the gradual loss of efficacy of VIM-DBS over time [12, 25]. The need for the continuous adjustment of the stimulation parameters during the follow-up period was likely the result of tolerance. With the progression of ET, the difficulty to control tremor is associated with a severe limb action tremor in these patients with already high scores at baseline [43]; moreover, the loss of effectiveness might be corrected by modulating the synchronized oscillatory cerebellothalamocortical pathway induced by high-frequency stimulation of the VIM [12]. Some investigators have reported that applying stimulation during waking hours or alternating stimulation protocols without increasing the stimulation strength can improve tolerance [25, 40].

### 4.3. Adverse Effects

Among the included studies, the incidence of stimulation-related AEs, surgery-related AEs, and device-related AEs was analyzed. Among the three types of AEs, the incidence of stimulation-related AEs (23.6%) was the highest, and these types of AEs were usually mild and easily improved by adjusting the stimulation parameters. Consistent with previous reports [44, 45], dysarthria, disequilibrium, motor disturbances, and paresthesia were the most common AEs [46]. Our analysis showed that the surgical-related AEs included infections (3.4%), asymptomatic bleeding (2.9%), intraoperative intracerebral hemorrhage (2.4%), and wound dehiscence (2.6%). Moreover, postoperative infection, hemorrhagic complications, pneumonia, and death associated with DBS are rare but often serious [13, 47]. Device-related AEs were common and bothersome after DBS of the VIM. In different reports, the complication rate ranged between 6.7% and 49% and often required additional surgery [48–51]. In our study, the device-related AE rate was 6.4%, and these types of AEs mainly included lead fracture (5.3%) and lead repositioning (3.8%). The device-related AER significantly decreased after 2003 [48]. Our rates were similar to those reported in the literature, and most included studies were published after 2003. We are convinced that the key factors responsible for lowering the complication rates of VIM-DBS are technical and hardware-related improvements and surgeon experience.

## 5. Limitations of the Study

Our meta-analysis had some limitations. First, most included studies were observational studies, and only four studies were RCTs, which has a certain impact on the quality of the incorporated resulting report. Larger randomized trials and prospective studies are required. Second, the potential prognostic factors are predicted through a univariate regression analysis rather than a multivariate regression analysis due to incomplete information in the included studies, such as follow-up time and disease duration. Thus, to evaluate the predictive factors of DBS using a more advanced method, authors reporting clinical trials should provide comprehensive data. Third, regarding the summary of tremor characteristics, all conclusions are based on a small sample, and more studies including an analysis of tremor characteristics are needed. Finally, regarding the methodology, our review was limited to the English literature and excluded some old publications that could not be retrieved.

## 6. Conclusions

DBS is an effective and safe treatment for patients with ET, but we need to be aware of the AEs. The efficacy was not affected by the body distribution of tremor, age at surgery, and disease duration. Moreover, VIM-DBS could be an effective strategy for ET patients with rest tremor, and the efficacy was similar not only between midline and extremity symptoms but also between postural and kinetic tremor. Lower preoperative FTM-TRS scores likely indicate larger improvements, and the effect of VIM-DBS declines over time. The age at surgery and disease duration may be prognostic factors of DBS in ET, but this hypothesis could not be confirmed based on our data. Clinical studies involving large samples of ET patients and prospective, randomized clinical studies are warranted to predict the potential prognostic factors in the future.

## Figures and Tables

**Table 1:** Details of studies included in a meta-analysis of DBS in treatment of ET.

Authors & year	No. of Pts	Age in Yrs (range)	Duration of ET in Yrs (range)	Site of lesion	Follow-up in Mons	Unil/Bil	Voltage (V)	Pulse widths (*μ*s)	Preoperative tremor scores	Postoperative tremor scores	Type of TRS	Percentage change in any TRS (%)
Koller W.C et al. 2001	25	72.3 ± 8.9	33.3 ± 15.4	VIM	40.2 ± 14.7	Unil	3.6 ± 1.3	99.6 ± 45.7	6.5 ± 1.6	1.4 ± 1.4	FTM-TRS	78.5
Ondo et al. 2001	13	71.5 ± 4.9	NS	VIM	3	Bil	NS	NS	6.7 ± 0.9	1.3 ± 1.2	MTRS	80.6
Pahwa R et al. 2001	17	73.1 (62–82)	NS	VIM	3.1	Unil	NS	NS	61.6 ± 13.2	30.5 ± 10.8	FTM-TRS	50.5
Fields J.A et al. 2003	40	71.7 ± 8.84	18.14 ± 12.88	VIM	12	Unil	L: 3.18 ± 0.53 R: 3.24 ± 0.75	L: 100.29 ± 32.49 R: 84.00 ± 25.10	19.35 ± 6.85	8.45 ± 4.13	FTM-TRS	56.3
Papavassiliou E et al. 2004	37	66.±13.6	NS	VIM	26 ± 16.2	21 Unil/16 Bil	2.7 ± 0.9	98.5 ± 27	19.3 ± 5.1	9.1 ± 6.2	FTM-TRS	52.8
Kuncel A.M et al. 2006	14	66.9 ± 17.2	NS	VIM	21.8 ± 17.9	NS	2.99 ± 0.83	87.85 ± 21.1	2.3 ± 0.7	0.04 ± 0.1	FTM-TRS	98.3
Van den Wildenberg WP et al. 2006	10	61.7 ± 11.8	13.5 ± 6.84	VIM	NS	NS	NS	NS	47.3 ± 23.42	30 ± 16.5	FTM-TRS	36.6
Pahwa R et al. 2006	22	70.6 ± 5.3	NS	VIM	60	3 Unil/20 Bil	3.6	111	23.9 ± 7.8	10 ± 4.9	FTM-TRS	58.2
Blomstedt et al. 2007	19	66 ± 11.1	NS	VIM	13	Unil	1.8 ± 0.7	68 ± 14	57.6 ± 19.2	29.2 ± 14.2	ETRS	49.3
Ellis TM et al. 2008	5	66 ± 11.1	29.6 ± 14.4	VIM	14 ± 5.33	NS	NS	NS	40.4 ± 4.41	16 ± 9.82	FTM-TRS	60.4
Zhang K et al. 2010	34	58.±12.8	22.1 ± 13.1	VIM	56.9	23 Unil/11 Bil	2.44 ± 0.89	81.9 ± 18.2	3.27 ± 0.87	0.64 ± 0.75	FTM-TRS	80.4
Morishita. et al. 2010	19	64.±12.3	26.3 ± 19.1	VIM	6	Unil	NS	ND	14.4 ± 4.43	3.63 ± 2.73	MTRS	74.8
Graft-Radford et al. 2010	31	66.4 ± 10.7	31.5 ± 20.6	VIM	6	22 Unil/9 Bil	Unil: 2.7 Bil (L): 2.7 Bil (R): 2.9	Unil: 102 Bil (L): 7 8 Bil (R): 9 3	58.2 ± 14.8	23 ± 12.6	FTM-TRS	60.5
Barbe et al. 2011	21	65 ± 14	27 ± 18	VIM	2.5	3 Unil/ 20 Bil	NS	NS	10.93 ± 7.53	7.39 ± 5.17	FTM-TRS	32.4
Vassal F et al. 2012	7	NS	NS	VIM	46.3 ± 28.7	NS	NS	NS	3.8 ± 0.3	0.37 ± 0.75	FTM-TRS	90.3
Zahos P.A. et al. 2013	7	66.6 ± 10.6	12.6 ± 6.6	VIM	10.1 ± 4.3	3 Unil/4 Bil	NS	NS	31.8 ± 13	7.7 ± 4.5	ETRS	75.8
Felicitas Ehlen et al. 2014	13	69.3 ± 9.43	15.77 ± 13.50	VIM	36.24 ± 33.69	Unil	3.13 ± 1.37	70.00 ± 14.77	16.15 ± 7.2	5.08 ± 5.12	UPDRS^a^	68.5
Rodríguez, C et al. 2016	14	61 ± 2.5	25 ± 10.5	VIM	6	3 Unil/11 Bil	90.0 ± 15.0	90.0 ± 15.0	63.3 ± 9.9	16.8 ± 11.2	FTM-TRS	73.5
Isaacs D.A et al. 2018	7	60.2 ± 11.6	NS	VIM	NS	NS	NS	NS	17.6 ± 4	6.9 ± 4	FTM-TRS	60.8
Paschen et al. 2018	21	NS	NS	VIM	NS	Bil	NS	NS	55.0 ± 3.7	20.9 ± 2.5	TRS^b^	62.0
Barbe M.T et al. 2018	13	58.9 ± 17.0	23.5 ± 17.8	VIM, PSA	12	NS	NS	NS	47.4 ± 7.9	23.8 ± 6	FTM-TRS	49.8
Akram H et al. 2018	5	63.8 ± 10.2	9.8 ± 2.0	VIM	23.6 ± 9.4	Unil	60 ± 0	60 ± 0	81.6 ± 17.6	48 ± 17.9	FTM-TRS	41.2
Fenoy, A.J et al. 2018	20	62.8 (18-81)	27.5 ± 15.4	VIM	12	2 Unil/18 Bil	2.9 ± 1.2	86 ± 33	2.1 ± 0.74	0.4 ± 0.5	ETRS	81.0
Paschen et al. 2019	20	66.6 ± 1.8	37.0 ± 3.8	VIM	13.1 ± 1.9	Bil	L: 2.44 ± 0.2 R: 2.47 ± 0.2	L: 66 ± 2.8 R: 63 ± 2.1	56.3 ± 3.7	20.9 ± 2.7	ETRS	62.9
Morishit T et al. 2019	3	70.7 ± 6.6	6 ± 4.32	VIM	16 ± 5.7	Unil	3.1 ± 0.36	110 ± 14.1	28.0 ± 2.94	15.67 ± 2.05	ETRS	44.0
Reich M et al. 2017	2	73	NS	VIM	NS	Bil	NS	NS	10.2 ± 9.5	4.8 ± 4.2	FTM-TRS	52.9
Pooled	439	—	—	—	—	—	—	—	—	—	—	61.3, *P* < 0.001

NS: not specified; Pts: patients; ET: essential tremor; Unil: Unilateral; Bil: Bilateral; TRS: Tremor Rating Scale; ETRS: Essential Tremor Rating Scale; Mons: Months. FTM: the Fahn-Tolosa-Marin; MTRS: Modified Tremor Rating Scale; UPDRS: the Unified Parkinson Disease Rating Scale. Unless otherwise stated, values are presented as the mean ± SD. ^a^Tremor intensity was defined using the sum score of UPDRS subitems 20 and 21 (tremor at rest, action, and postural). ^b^The detailed type of TRS was not definite in Paschen et al. 2018.

**Table 2:** Statistical difference of patient characteristics among laterality and stimulation parameter (pulse width and voltage) subgroups^a^.

	Laterality			Voltage (V)			Pulse widths (*μ*s)		
	Unilateral	Bilateral	*P* value	≥3.5	<3.5	*P* value	60-90	90-120	*P* value
Age at surgery (years)	69.74 ± 0.765	67.529 ± 2.201	0.115	67.868 ± 3.959	66.93 ± 1.094	0.375	65.820 ± 1.480	68.253 ± 2.617	0.782
Disease duration (years)	18.654 ± 3.488	37 ± 0.85	0.022^b^	29.053 ± 4.149	21.273 ± 5.024	0.247	26.092 ± 4.235	20.496 ± 5.405	0.205
Follow-up (months)	41.528 ± 18.174	22.787 ± 11.416	0.224	65.071 ± 26.166	42.599 ± 16.243	0.245	42.206 ± 17.054	38.828 ± 8.171	0.447

^a^The data are represented by “mean ± standard error”. ^b^The disease duration of bilateral procedure is almost missing, and only one data exists, so there are large biases in the significant comparison.

**Table 3:** Summary of critical appraisal of included studies using the Newcastle-Ottawa Scale for assessing the quality of observational studies.

Study	Selection	Comparability	Outcome
Koller W.C et al. 2001	★★	★	★
Ondo et al. 2001	★★★	★	★★
Pahwa R et al. 2001	★★★	★★	★★
Fields J.A et al. 2003	★★		★
Papavassiliou E et al. 2004	★★		★
Kuncel A.M et al. 2006	★★	★★	★★
Van den Wildenberg WP et al. 2006	★★	★	★
Pahwa et al. 2006	★		★
Blomstedt et al. 2007	★★	★	★
Ellis TM et al. 2008	★★	★	★★
Zhang K et al. 2010	★★		★
Morishita T et al. 2010	★		★
Graff-Radford J et al. 2010	★	★	★
Barbe et al. 2011	★		★★
Vassal F et al. 2012	★★	★	★★
Zahos P.A. et al. 2013	★★	★	★★
Felicitas Ehlen et al. 2014	★★	★	★
Rodríguez, C et al. 2016	★		★★
Isaacs D.A et al. 2018	★		★★
Paschen S et al. 2018	★		★
Barbe M.T et al. 2018	★★	★	★★★
Akram H et al. 2018	★★		★★
Fenoy, A.J et al. 2018	★★★	★	★★
Paschen S et al. 2019	★★		★★★
Morishita T et al. 2019	★★		★★
Reich M et al. 2017	★		★★
Hubble J.P. et al. 1996	★★	★	★
Koller W.C et al. 1999	★★	★	★
Taha J M. et al. 1999	★★	★	★★
Rehncrona S et al. 2003	★★	★	★★
Lee J Y.K. et al. 2005	★★	★	★
Törnqvist A. L et al. 2007	★★	★	★★
Lind G et al. 2008	★★	★	★★
Blomstedt P et al.2010	★★	★	★
Baizabal Carvallo JF et al. 2012	★★	★	★★
Carballal C.F. et al. 2013	★★	★	★★
Borretzen M.N. et al. 2014	★★	★	★
Baizabal Carvallo JF et al. 2014	★★	★	★
Hariz G-M et al. 2015	★★	★	★
Verla T. et al. 2015	★★	★	★
Sharma V.D et al. 2015	★★	★	★★
Silva D et al. 2016	★★	★	★★
Klein J et al. 2017	★★	★	★★
Wharen R E. et al. 2017	★★	★	★
Chen T et al. 2018	★★	★	★★
Koller W.C et al. 1999	★★★	★★	★★
Kuncel A.M et al. 2006	★★★	★★	★★
Felicitas Ehlen et al. 2014	★★★	★★	★
Barbe M.T et al. 2018	★★★	★★	★★

Each of these three categories has further subcategories and gives stars. The studies with the maximum number of stars are of higher quality than those with fewer stars. Empty cells show that no stars are available for this category.

**Table 4:** Summary of all adverse effects.

Study	Adverse event		
	Stimulation	Surgical	Device
Hubble J.P. et al. 1996	Paresthesia (10), dysarthria (1), headache (2), face-arm pain (1), right-sided weakness (3), face weakness (1), decreased range of motion left shoulder(1)	0	0
Koller W.C et al. 1999	Mild paresthesia (24), mild headache (9), mild dysarthria (7), mild paresis (6), attention/cognitive deficits (2), gait disorder (2), facial weakness (2), dystonia (1), hypophonia (1), nausea (1), mild depression (1), dizziness (1)	Subdural hematoma (1), intraparenchymal hemorrhage (1), asymptomatic bleeds (3), seizures (1)	Loss of effect (8), lead replacement (2), devices explanted (2), reprogrammed (1), broken lead (1), lead extension replacements (2), IPG replacement (1)
Taha J M. et al. 1999	Disequilibrium (7), mild short-term memory loss (1), mild shock (4), dysarthria (7)	0	0
Koller W.C et al. 2001	Paresthesia (21), headache (15), paresis (6), dysarthria (4), nausea (4), disequilibrium (3), facial weakness (3), gait disorder (2), dystonia (2), mild attention/cognitive deficit (2), dizziness (2), hypophonia (1), anxiety (1), depression (1), syncope (1), vomiting (1), shocking sensation (1), drooling (1)	Asymptomatic bleeds (3), postoperative seizures (1)	Lead replacement (7), lead reposition (3), extension wire replaced (3), IPG replaced (4), entire system explanted (1)
Ondo et al. 2001	Paresthesia (3), headache (5), dysarthria (7), neck pain (2), mouth pain (1), increased saliva (1), balance and gait difficulty (10)	0	0
Pahwa R et al. 2001	Headache (9), paresthesia (10), dysarthria (1), disequilibrium (1), dizziness (2)	Seizures (1)	0
Rehncrona S et al. 2003	0	0	Lead fracture (1)
Lee J Y.K. et al. 2005	Hand tingling (3)	Temporary erythema of the incision (1)	Lead fracture (1), electrode migration (1)
Kuncel A.M et al. 2006	Dysarthria (9), posturing (7), jaw deviation (3), eye closure (2), voice affected (2)	0	0
Blomstedt et al. 2007	0	0	IPG exchange (12), lead fracture (6)
Törnqvist A. L et al. 2007	0	Infections (2)	Lead fracture (1)
Ellis TM et al. 2008	0	0	Lead fracture (1), lead migrated (1)
Lind G et al. 2008	Speech disorder (3), balance and gait difficult (2)	Infections (2)	Battery replacement (6)
Blomstedt et al. 2010	Aphasia (8), clumsy (1)	0	0
Baizabal Carvallo JF et al. 2012	0	Infections (3)	Misplacements (4), migrations (5), fractures (5)
Zahos P.A. et al. 2013	0	Wound dehiscence (2)	Lead fracture (1)
Carballal C.F. et al. 2013	Headache (9), paresthesia (6), dysarthria (17), dizziness (5), reduced balance (4)	Infections (1)	0
Borretzen M.N. et al. 2014	Dysarthria (17), headache (9), paresthesia (6), abnormal taste (8), dizziness (5), discomfort tongue (4), reduced balance or coordination (4)	0	0
Baizabal Carvallo JF et al. 2014	Incoordination (7), dysarthria (6)	0	Vasovagal reaction (1)
Hariz G-M et al. 2015	Headache (1), voice affected (5), deterioration of balance (4), tiredness (1), tearful (1), felt discomfort (1)	0	0
Verla T. et al.2015	0	Hemorrhagic complication (10), infection (20), pulmonary embolism (4), pneumonia (16)	Lead revision (2), generator revision (7)
Sharma V.D et al. 2015	Incoordination (1), dysarthria (1), paresis (1), asthenia (1), reduced balance (1)	0	0
Silva D et al. 2016	Paresis (2), dysarthria (6), transient cognitive alter (1), facial numbness (1)	Hemorrhage (1), infections (1)	0
Klein J et al. 2017	0	Infections (1)	Wound revision (3), electrode dislocation (1)
Wharen R E.et al. 2017	Speech disturbances (12), gait/postural disorder (5), cognitive changes (8), dysphagia (2), tinnitus (1), shocking or jolting sensation (3), discomfort (17), headache (8), paresis (1), dystonia (2), dysarthria (1), hemiparesis (1)	Seizures (1), intracranial hemorrhage (3), wound dehiscence (4), infections (5), pocket hematoma (2)	Misplaced lead (6), IPG malfunction (4), extension malfunction (6), battery check (9)
Barbe M T et al. 2018	Right hemiparesis (1), dysarthria (11), aphasia (1), nausea (1)	Intracerebral hemorrhage (1)	0
Chen T et al. 2018	Mental status change (9), speech disturbance (7), balance or gait disturbance (6), speech & balance disturbances (5)	Hemorrhage (1), wound breakdown (1)	0

The number in brackets means the number of AE events.

## Abbreviations

DBS: Deep brain stimulation
AEs: Adverse effects
ET: Essential tremor
VIM: Ventral intermediate nucleus
TRS: Tremor rating scale
AERs: Adverse effect rates
RCTs: Randomized controlled trials
OR: Odds ratio
FTM: Fahn-Tolosa-Marin.

## Supplementary Materials

Supplementary Materials Supplementary 1: comparison of the improvement in midline and extremity symptoms following DBS among ET patients. Supplementary 2: comparison of the improvement in rest and action tremor following DBS among ET patients. Supplementary 3: comparison of the improvement in postural and kinetic tremor following DBS among ET patients. Supplementary 4: incidence of common adverse effects. Supplementary 5: incidence of rare adverse effects.
