# Supplementary material for: Outcomes and Adverse Effects of Deep Brain Stimulation on the Ventral Intermediate Nucleus in Patients with Essential Tremor
Source: Neural Plast. 2020 Aug 1;2020:2486065. doi: 10.1155/2020/2486065 (PMC7416257; doi:10.1155/2020/2486065)
Supplement: Supplementary Materials — Supplementary 1: comparison of the improvement in midline and extremity symptoms following DBS among ET patients. Supplementary 2: comparison of the improvement in rest and action tremor following DBS among ET patients. Supplementary 3: comparison of the improvement in postural and kinetic tremor following DBS among ET patients. Supplementary 4: incidence of common adverse effects. Supplementary 5: incidence of rare adverse effects. [file 2486065.f1.doc]

**APPENDIX**

( The following is our supplementary material to the relevant data, not in the text)

**Supplementary 1. Comparison of the improvement in midline and extremity symptoms following DBS among ET patients**

| **Study name** | **No. of**  **patients** | **Midline(head/voice）** | | | **Extremities(arms/legs)** | | | **P value** |
| --- | --- | --- | --- | --- | --- | --- | --- | --- |
|  |  | **Preoperative**  **scores** | **Postoperative**  **scores** | **% Improvement** | **Preoperative**  **scores** | **Postoperative**  **scores** | **% Improvement** |  |
| **Blomstedt P.et.al,2007** | 19 | head:1.5±1.9  voice:0.7±1.0 | head:0.5±0.8  voice:0.4±0.8 | 54.5  (combined) | ipsilateral:5.1±3.1  contralateral:6.8±3.1 | ipsilateral:5.0±2.7  contralateral:1.2±1.7 | 71.0  (combined) | 0.296 |
| **Fenoy.A.J. et.al,2018** | 20 | head:2.1±0.74 | head:0.4±0.5 | 81.0 | left:2.97±0.6  right:3.0±0.6 | left:1.18±0.2  right:0.9±0.6 | 65.0  (combined) | 0.260 |
| **Isaacs.D.A.et.al,2018** | 7 | 2.6 ±1.0 | 1.7±1.3 | 34.6 | left:7.1±2.3  right:7.9±3.7 | left:2.7±1.8  right:2.4±2.0 | 65.7  (combined) | 0.253 |
| **Ondo.W.et.al,2001** | 6 | head:1.8±1.2  voice:1.2±0.9 | head:1.0±0.7  voice:0.5±0.5 | 51.3  (combined) | upper:6.7±0.9  lower:2.3±1.1 | upper:1.3±1.2  lower:0.5±0.5 | 79.4  (combined) | 0.318 |
| **Overall** | 52 | **OR=0.716**, 95%CI:0.307-1.670 | | | | | | 0.440 |

**Supplementary2. Comparison of the improvement in rest and action tremor following DBS among ET patients**

| **Study name** | **No. of**  **patients** | **Rest tremor** | | | **Action tremor** | | | **P value** |
| --- | --- | --- | --- | --- | --- | --- | --- | --- |
|  |  | **Preoperative**  **scores** | **Postoperative**  **scores** | **% Improvement** | **Preoperative**  **scores** | **Postoperative**  **scores** | **% Improvement** |  |
| **Blomstedt P.et.al,2007** | 19 | 0.8 ±1.4 | 0.0±0.0 | 100 | 3.5±1.0 | 1.1±1.5 | 68.5 | 0.052 |
| **Morishita T.2010** | 19 | 0.28 ± 0.67 | 0.05 ± 0.23 | 82.1 | 2.61 ± 0.85 | 0.68 ± 1.12 | 73.9 | 0.544 |
| **Zahos P.A.2013** | 7 | 2.0 ± 1.7 | 0.0 ± 0.0 | 100 | 2.8 ± 1.6 | 0.3 ± 0.7 | 89.2 | 0.558 |
| **Overall** | 45 | **OR=2.759**,95%CI：0.768-9.913 | | | | | | **0.120** |

**Supplementary3.Comparison of the improvement in postural and kinetic tremor following DBS among ET patients**

| **Study name** | **No. Of patients** | **Tremor type** | **Preoperative scores** | **Postoperative scores** | **% Improvement** |
| --- | --- | --- | --- | --- | --- |
| **KuncelA.M et.al.,2006** | 14 | postural | 2.3±0.7 | 0.04±0.1 | 98.3 |
| **Blomstedt P et.al., 2007** | 14 | postural | 2.5±1.4 | 0.2±0.4 | 92.0 |
| **Morishita et.al.,2010** | 19 | postural | 2.33±1.44 | 0.11±0.32 | 95.3 |
| **combined** | 52 | postural | ------ | ------ | 94.2 |
| **Graft-Radford.J et.al.,2010** | 31 | kinetic | 17.2±4.1 | 9.4±4.5 | 45.3 |
| **Zahos P.A. et.al., 2013** | 7 | kinetic | 2.8±1.6 | 0.3±0.7 | 89.3 |
| **Pahwa R et.al., 2001** | 17 | kinetic | 19.2±3.6 | 11.6±3.3 | 39.6 |
| **combined** | 55 | kinetic | ------ | ------ | 46.5 |
| **Subgroup comparison** | 52 vs 55 | postural vs kinetic | P = 0.219 | | |

**Supplementary 4. Incidence of common adverse effects**

| **Common Adverse Effects** | | |
| --- | --- | --- |
| **Stimulation(23.6%)** | **Surgical(6.4%)** | **Device(11.5%)** |
| dysarthria (10.5%) | infections(3.4%) | lead fracture(5.3%) |
| headache (6.7%) | asymptomatic bleeds(2.9%) | battery replacement(4.3%) |
| hemiparesis/paresis(6.3%) | wound dehiscence(2.6%) | lead reposition(3.8%) |
| paresthesia (6.3%) | intracerebral hemorrhage(2.4%) | battery check(3.6%) |
| dizziness(4.9%) | seizures(2.3%) | extension wire replaced(3.6%) |
| voice effected(4.3%) | vasovagal reaction(2.3%) | IPG replacement(3.2%) |
| transient altered mental status/depression/anxiety(4.2%) | temporary erythema(2.2%) | electrode migration(3.2%) |
| mild attention/cognitive deficit/transient cognitive alter(3.9%) |  |  |
| shock(3.6%) |  |  |
| nausea(3.5%) |  |  |
| asthenia(3.3%) |  |  |
| facial weakness(3.3%) |  |  |
| deterioration of balance(3.3%) |  |  |
| pain(3.3%) |  |  |
| reduced balance or coordination(3.2%) |  |  |

**Supplementary 5. Incidence of rare adverse effects**

| **Rare Adverse Effects** | | |
| --- | --- | --- |
| **Stimulation-related** | **Device-related** |  |
| Jaw deviation(3.1%) | dystonia(2.6%) | wound revision(2.9%) |
| disequilibrium(3.1%) | hypophonia(2.3%) | extension malfunction(2.8%) |
| incoordination (3.1%) | increased saliva(2.3%) | lead replacements(2.4%) |
| balance and gait difficult(3.0%) | syncope(2.2%) | devices explanted(2.4%) |
| hand tingling(3%) | drooling(2.2%) | IPG malfuncction(2.4%) |
| gait disorder(2.9%) | vomit(2.2%) | lead migrated(2.3%) |
| speech disorder(2.9%) | facial numbness(2.2%) | entire system explanted(2.2%） |
| aphasia(2.8%) | tiredness(2.2%) | electrode dislocation(2.2%) |
| posturing(2.7%) | tearful(2.2%) | reprogrammed(2.2%) |
| abnormal gait(2.6%) | felt discomfort(2.2%) |  |
| eye closure(2.6%) | increased saliva(2.1%) |  |
